# Characterization of Fruit Sorbet Matrices with Added Value from *Zizyphus jujuba* and *Stevia rebaudiana*

**DOI:** 10.3390/foods11182748

**Published:** 2022-09-07

**Authors:** Teodora Petkova, Pavlina Doykina, Iordanka Alexieva, Dasha Mihaylova, Aneta Popova

**Affiliations:** 1 Department of Catering and Nutrition, Economics Faculty, University of Food Technologies, 4002 Plovdiv, Bulgaria; 2 Department of Biotechnology, Technological Faculty, University of Food Technologies, 4002 Plovdiv, Bulgaria

**Keywords:** fruit, healthy dessert, vegan nutrition, health-promoting, frozen

## Abstract

Sorbets are healthy ice cream alternatives and desired frozen desserts by vegetarians and vegans. This study focuses on assessing the effects of sorbet recipe alteration through the addition of different percentages of *Zizyphus jujuba* powder. *Stevia rebaudiana* was used as a sugar substituent. A control sample and five variables were developed. Peaches from the “Laskava” (native Bulgarian) variety were used as the main ingredient. The new sorbet matrices were characterized based on their moisture and ash content, overrun, melting behavior, melting rate, water holding capacity, pH, nutritional data, soluble solids content, titratable acidity, vitamin C content, total phenolic content and antioxidant activity. The microbial load and CIELAB color of the sorbet alternatives was also evaluated. A sensory evaluation revealed the most preferred variant. Panelists evaluated the appearance (*n* = 6), aroma (*n* = 5), flavor (*n* = 5), mouthfeel (*n* = 7), and aftertaste (*n* = 5). The soluble solids content varied from 17.50 to 33.03%, the ash content from 0.36 to 5.21%, the moisture content from 63.77 to 80.21%. The studied sorbet matrices have an overrun in the range from 8.11 to 12.32%. Results showed that a potential for the development of peach sorbet matrices with added value and a reasonable consumer acceptability exists. Further research can perfect the recipe and provide a reference for other frozen desserts.

## 1. Introduction

Frozen desserts are especially popular and preferred in high temperatures. Different types of ice creams are most often consumed by all age groups. Sorbets are a healthier alternative to ice cream, very suitable for vegans, vegetarians, and consumers who would like to lower their daily calory intake [1]. Sorbets provide an opportunity for the incorporation of the whole fruit even if it is marked with some damages, and labelled unsuitable for market sale. Their easy preparation through the use of technology is another advantage for their broader consumption and market availability. Frozen desserts have been studied for a long time, and the ongoing scientific interest in their characterization is evident from the availability of papers published on the subject [2,3]. The contemporary consumer, with his increased health consciousness, is in a constant search for new health-promoting value-added products. Sorbets may act as healthy frozen snacks with numerous taste possibilities depending on the fruit added in the recipe. New product development is a trending research area focusing on various products, i.e., healthier ice cream alternatives [4] products with longer shelf life, or those with added value from specific ingredients (inulin, antioxidants, vitamins, amino acids, among others). Sorbets may even be labelled bio if appropriate ingredients are used. The food processing industry has long anticipated a zero-waste production cycle, and waste valorization through the retrieval of high-added-value compounds [5,6]. The jujube (*Ziziphus jujuba*) fruit is recognized as a dietary value-added product due to its anti-allergy, anti-cancer, anti-inflammation, anti-aging, anti-hyperglycemic, anti-hyperlipidemic, and neuroprotective activities [7,8]. This fruit is very well known and consumed in China [9], but remains relatively unknown and underutilized in Europe. Stevia, on the other hand, has gained worldwide popularity as a natural sweetener with functional aspects [10]. Peaches, with their great cultivar availability, are a popular and sought-after dessert fruit. They are usually consumed fresh, canned or as a component of baked goods. They contain numerous health promoting properties, i.e., cardiovascular health, brain function improvement, non-communicable diseases improvement [11,12]. Due to the above-mentioned properties and their traditional use in culinary technology, peaches can be seen as suitable for being the main ingredient in frozen desserts.

This study focuses on assessing the effects of sorbet recipe alteration through the addition of different percentages of *Zizyphus jujuba* powder. *Stevia rebaudiana* was used as a possible sugar substituent. The sorbet matrices were characterized based on their moisture and ash content, overrun, melting behavior, melting rate, water holding capacity, pH, nutritional data, soluble solids content, titratable acidity, vitamin C content, total phenolic content and antioxidant activity. The microbial load and CIELAB color of the sorbet alternatives was also evaluated. A sensory evaluation revealed the most preferred variant.

## 2. Materials and Methods

### 2.1. Materials

Fresh peach samples of the “Laskava” variety were provided by the Fruit Growing Institute (Plovdiv, Bulgaria). Sugar and citric acid used in the current study were purchased from a local “Lidl” store (Plovdiv, Bulgaria), while stevia was purchased at a local “dm drogerie” store in Plovdiv, Bulgaria. Dry jujube granules were imported from the UK and purchased from DD Ltd., Sofia, Bulgaria. The 50 g packages were produced from “Abakus foods”, London, UK. Tap water was used for all sorbet variations.

### 2.2. Preparation of Sorbet Variations

The sorbet matrices were prepared in laboratory conditions at the University of food technologies. Table 1 provides information about the percentage distribution of the ingredients used to prepare the variations.

**Figure 1:**
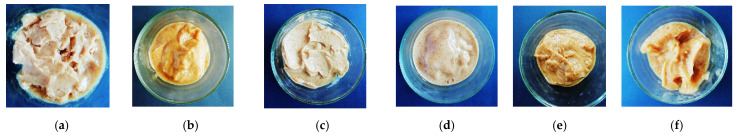
Sorbet matrices. (**a**) control; (**b**) SR5; (**c**) SR10; (**d**) SR15; (**e**) SZJ; (**f**) SSR.

The fresh peaches were puréed using Muhler Nutritional blender MNB-688. Jujube granules were powdered with Tefal GT110838 grinder. All the necessary ingredients were pre-mixed and placed in a “Delimano” ice cream maker until the mixture reaches a creamy structure and a temperature range of −2 to −6 degrees centigrade. The variations (Figure 1) were then placed into containers according to their further usage, and stored in a freezer.

### 2.3. Ash and Moisture Content

Ash content was determined by burning in a muffle furnace according to AOAC 945.46. The moisture content of the sorbet variations was measured using an infrared moisture analyzer PMB 53 (Adam Equipment Inc., Oxford, UK).

### 2.4. Total Soluble Solids (TSS), pH and Titratable Acidity

TSS (%) were measured using a digital handheld refractometer (Opti Brix 54, Bellingham + Stanley, Kent, UK). The pH was quantified using an Orion 2 Star pH Benchtop (Thermo Scientific, Singapore) with the electrode standardized to pH 4.0 and 7.0 Sigma buffers. Titratable acidity was determined with the use of the potentiometric method, and a pH meter Orion 2 Star pH Benchtop (Thermo Scientific, Singapore).

### 2.5. Nutritional Data

The calculation method was used in order to present the nutritional data of 100 g finished product. Values for each purchased ingredient were based on specifications obtained from suppliers. The nutritional value of the “Laskava” peach lied on previous research [13].

### 2.6. Overrun, Melting Rate and Melting Behavior

Overrun (OR, %) was determined as described by Marshall et al. [14] and calculated according to the Equation:

OR (%) = (weigh of sorbet mix − weigh of sorbet/weigh of sorbet) × 100(1)

Melting rate (g/min) was evaluated as described by Balthazar et al. [15]. The mass of melted sorbet was recorded every 5 min for a period of 120 min aiming at obtaining a sigmoidal curve representing the kinetics of the melting process. Melting behavior was assessed through an image analysis where, at fixed intervals of 10 min for a period of 90 min a photograph, using a Huawei P Smart 2019 camera phone, of the sorbet variation was taken in standard room conditions (22 °C) in order to present its ability to retain its shape during melting.

### 2.7. Water-Holding Capacity

Water holding capacity (WHC) was defined as originally described by Raungrusmee and Anal [16] and modified by Mihaylova et al. [17].

### 2.8. CIELAB Color Spectra of Sorbets Variations

A PCE-CSM 2 (PCE-CSM instruments, Meschede, Deutschland) with a measuring aperture of 8 mm was used to analyze the color parameters (L, a, b, c, h).

### 2.9. Determination of the Vitamin C Content

The vitamin C content was measured using the dichlorophenolindophenol titration as described by Popova [18].

### 2.10. Determination of Total Polyphenolic Content

The TPC was analyzed following a modified method of Kujala et al. [19]. The TPC was expressed as mg gallic acid equivalents (GAE) per 100 g product after being vortexed, left for 5 min at 50 °C and measured at 765 nm for its absorbance.

### 2.11. Determination of Antioxidant Activity

The antioxidant activity was characterized with the use of four contemporary assays- DPPH^•^ radical scavenging assay, as described by Mihaylova et al. [20]; ABTS^•+^ radical scavenging assay, as published by Re et al. [21]; FRAP assay, as described by Benzie and Strain [22]; and the CUPRAC assay carried out according to the procedure of Apak et al. [23]. Trolox was used as a standard and the results were expressed as TEAC value (μM TE/100 g product).

### 2.12. Microscopic Imaging

The air bubbles were determined using USB Digital pocket microscope MX200-B with ×1000 LED magnification endoscope camera with a focus range of 1–9 mm.

### 2.13. Microbial Count

Potato Dextrose Agar plates were incubated at 30 °C and counted after 72 h. in order to determine yeasts and molds (YM). The aerobic mesophilic microorganisms (AMM) count was evaluated according to ISO 4833-1:2013 [24] using Plate Count agar as culture medium. Both YM and AMM were based on the spread-plate method. The results were expressed as Colony Forming Units (CFU)/mL.

### 2.14. Sensory Evaluation

Sensory analysis was performed at the University of Food Technologies as described by Mihaylova et al. [17]. Panelists evaluated the appearance (*n* = 6), aroma (*n* = 5), flavor (*n* = 5), mouthfeel (*n* = 7), and aftertaste (*n* = 5).

### 2.15. Statistical Analysis

Produced data were statistically analyzed with the use of MS Excel software. All assays were performed in at least triplicates in order to present the results as mean ± SD (standard deviation). Additional statistical analyses of the data were performed using one-way ANOVA and a Tukey-Kramer post hoc test (α = 0.05), as described by Assaad et al. [25].

## 3. Results and Discussion

Different parameters have been studied in order to better qualify the sorbet matrices. Information about the soluble solid content, titratable acidity, pH, ash content and moisture content of the control sample and sorbet variations is presented in Table 2.

The results concerning the pH of the sorbets show similarity not only among the studied variations, but also to other frozen desserts, such as frozen blackcurrant products [26] and passion fruit ice creams [26,27]. The lowest obtained pH value (control 3.37) does not correspond to the lowest TTA value (SR15 0.18). The moisture content is quite similar with the exception of the SSR variant where stevia was used. The other sorbet matrices have comparable moisture content to data published in previous papers [26]. The ash content decreased with the increased jujube content in the sorbets. The same tendency was observed when the TSS values were evaluated. The TSS values correspond to the ones documented about Acacia honey lime ice cream formulations [28].

Both jujube and peaches are reported to contain vitamin C [11,29], thus Table 3 visually presents the vitamin C content of the studied sorbet matrices.

It can be observed that the vitamin C content is significantly low, but the inclusion of more jujube increases its content. This may serve as proof that the jujube fruit powder contributes more to the vitamin C content compared to the fruit purée. Similar conclusions about the enhancing properties of fruit on the vitamin C content of ice creams have been made by other research teams [30].

The proximate nutritional information of the studied sorbet matrices is given in Table 4. When taking into account regulation (EC)1924/2006 [31] of the European Parliament and of the Council of 20 December 2006 about possible health claims only two of the formulations (SZJ and SSR) can account for with no added sugar, and contains naturally occurring sugars, while all formulations refer to low-fat.

When evaluating the protein content, it is evident from Table 3 that the studied sorbet matrices cannot contribute to the daily diet in terms of protein. These results are quite similar to the ones reporting the protein content of frozen blackcurrant products [26]. The inclusion of jujube powder was beneficial in terms of proteins for the recipe, although it could not significantly influence its content.

The same trend is observed for the carbohydrate content (an increase with the addition of more jujube powder). The established carbohydrate content is lower compared to traditional milk-containing ice cream recipes, and relatively the same with the commercially available sorbets.

All sorbet variations contribute to a small portion of the needed daily energy. The energy value, again, corresponds well to the variations available in stores.

**Figure 2:**
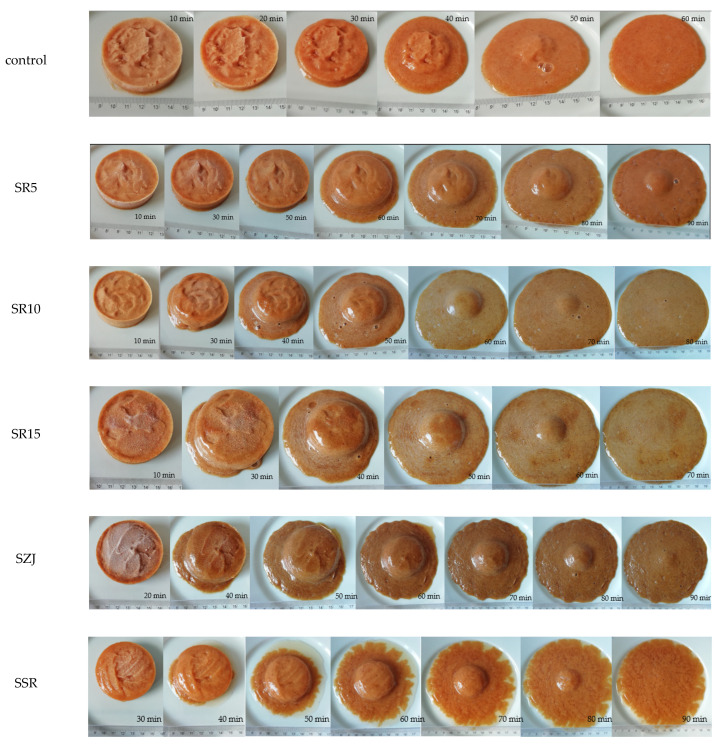
Melting behavior of sorbet matrices.

The melting behavior is an important factor for frozen desserts. The ability of the sorbet variations to hold their shape during melting is presented in Figure 2.

The control sample was completely melted for 60 min, while SR5 managed to withhold some of its shape even after the 90 min period. The SSR variation had the most distinct melting behavior compared to the others—a visible water outer ring formed during melting. The melting properties of the sorbets containing jujube did not differ significantly. Other authors [32] have also found that frozen dessert usually liquify for a period of 90 min.

**Figure 3:**
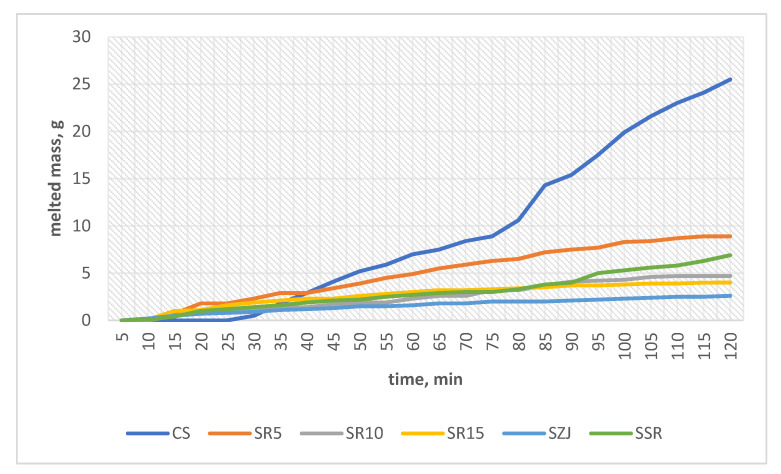
Melting rate of sorbet matrices.

Some papers [26] comment on the connection between the melting behavior and the overrun of ice creams. Thus, the overrun of the sorbet variations is given in Table 5 and the calculated melting rate is presented in Figure 3.

Most of the studied variations have a similar overrun; SR5 and SZJ have 8.11 ± 0.08 and 8.11 ± 0.08, respectively. Góral et al. [33] documented that a low overrun in frozen ice creams is most probably due to a low protein content. This statement is very well supported with the current results where the overrun in all sorbet matrices is significantly low compared to milk-containing ice creams [34]. Furthermore, Senanayake et al. [35] also validate that fruit ice creams are characterized with lower overrun. Additionally, a lower overrun is associated with a lower melting rate which is also very well supported with SR5. This particular sorbet has the lowest overrun and manages to withhold its shape the longest time.

The calculated melting rate corresponds well to the melting behavior of the studied samples. As it can be observed from the figure, the control sample has the highest melting rate. This may indicate that the added jujube powder aids positively in the melting kinetics of the sorbets. The slower the melting rate the better the melting behavior.

**Figure 4:**
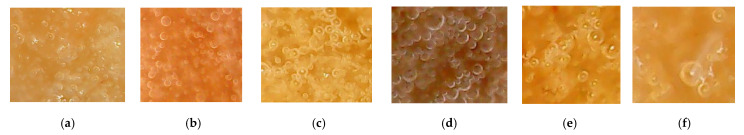
Sorbet matrices micrographs. (**a**) control; (**b**) SR5; (**c**) SR10; (**d**) SR15; (**e**) SZJ; (**f**) SSR.

Figure 4 shows the air bubbles size and distribution in the sorbet matrices immediately after preparation prior to additional freezing to harden the sample. The air bubbles are relatively the same in all sorbet variations.

The amount of air incorporated during freezing, affects the size of the ice crystals, with larger ice crystals and bubbles detected at lower overrun. It has to be noted though, the size of air bubbles fluctuates in the process of ice crystal formation [36].

As the color is perceived as an important characteristic for consumers, it is vital to determine the CIELAB color spectra of the studied sorbet matrices (Table 6).

All studied variations did not have much differences in color among each other. All samples were in the middle of the lightness range (42.13 ± 3.18 to 57.91 ± 4.27). This is the only indicator where results are statistically different. The a and b values show a tendency for the red and yellow chromaticity. As stated in previous papers [37], natural colorants are frequently perceived by lower “c” values and higher L values which is the case in the current study. Information about the color characteristics of sorbets exists in literature [38], but a comparison is not reasonable due to the differences in the ingredients used.

Table 7 reveals the microbial load of the studied sorbet matrices. All ingredients used could attribute to the microbial count of the samples. All variations were tested against yeast and molds as well as count of aerobic mesophilic microorganisms. All formulations can be considered safe for consumption.

At day one, the highest plate count was noticed in the SZJ sample, and lowest value in SR15, while the mold count of the formulations varied from 700 to 5400 CFU/mL. The highest microbial load was recorder in the SZJ sample which did not contain any sugar. This undoubtedly suggests that sugar has a positive effect on the microbial count of frozen desserts. Some authors suggest that this particular feature may be due to the lowering effect sugar has on the water activity of food samples [39,40]. The established acidic pH of the sorbets may also positively contribute to the safety of the finished product since acidic pH prevents microorganisms from growth. The addition of powdered ingredients may also be the reason for lower microbial load because water will bond with the powdered jujube and none will be left available for microorganisms to use.

The water-holding capacity of the studied samples is given in Table 8. Limited WHC implies that the samples cannot hold the non-chemically bound water in the food matrix, and thus they are unstable in response to gravity.

The WHC gradually decreases with the increased addition of jujube powder. The control sample has the highest WHC from all sorbet variations. Literature states that the addition of plant protein increases the water-holding properties and reduces the size of ice crystals in ice creams [41]. This statement does not comply with the current findings.

**Figure 5:**
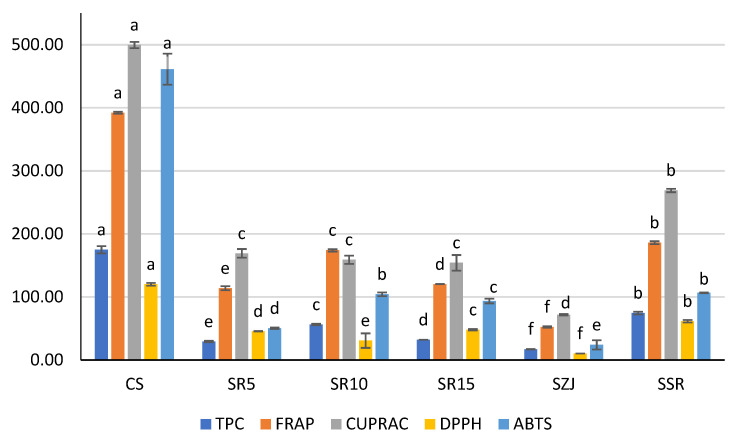
Total polyphenolic content (TPC) (mgGAE/100 g product) and antioxidant properties (µM/100 g product) of sorbet matrices. Different letters in the same column indicate statistically significant differences (*p* < 0.05), according to ANOVA (one-way) and the Tukey test.

The TPC (Figure 5) revealed that the control sample had the highest values. This indicates that the jujube powder is not the major contributor for the total polyphenolic content of the studied sorbet variations. Furthermore, the more the jujube content, the less the TPC and AOA. Contrary to the current fundings, coconut-enriched ice creams had increased total phenolics and minerals [42].

The results concerning the AOA confirm the abovementioned. All assays used in the AOA characterization (ABTS, FRAP, CUPRAC, DPPH) had their highest values in the control sample. The second highest results belong to the SSR sample, where again no jujube powder was present. The results shown in Figure 4 undoubtedly reveal a connection between the TPC and AOA.

A sensory evaluation (Table 9) made by a group of panelists aided in the characterization of the products in terms of their appearance (*n* = 6), aroma (*n* = 5), flavor (*n* = 5), mouthfeel (*n* = 7), and aftertaste (*n* = 5).

Not all sorbet variations were well accepted by the consumer panel. SZJ and SSR had an unpleasant flavor/aftertaste which makes them unsuitable for consumption no matter their potential added-value.

The panelists had to evaluate the appearance of the matrices prior to tasting them. When only appearance is considered, all variations were considered of natural ingredients due to their specific color in the orangey shade. They were described with homogenous consistency. The SR5 variation had the highest scores and future efforts should focus on perfecting the technology and recipe content of this particular sorbet. The jujube powder addition had a distinct flavor alteration compared to the peach control sorbet. The substitution of sugar with stevia can be ruled unsuccessful due to the bitter aftertaste. Future formulations may be developed with less stevia present in them. The SZJ variation was described as giving the perception of tongue numbing and having an unpleasant taste and aftertaste. Additionally, unpleasant mimics of the face confirmed the lack of acceptance in these two particular formulations.

Research on ice creams with extract addition reveals that the newly developed formulations may be better accepted by the consumer’s panel if additional information about the beneficial properties of the product is given [38].

## 4. Conclusions

Different peach sorbet matrices with added value from jujube fruit powder and stevia were characterized. Although physicochemical and chemical results showed potency, not all developed variations were with acceptable sensory characteristics. The replacement of sugar with stevia has significantly affected the acceptance of the finished product. However, stevia can be successfully incorporated in fruit sorbets after further recipe perfection. Jujube fruit powder had its best influence at 5% addition.

The developed sorbet matrices aimed at zero waste production cycle with the incorporation of both fruit skin and pulp in their technology. The current results provide reference for future frozen dessert development.

## Figures and Tables

**Table 1:** Sorbet matrices.

Sorbet Variations	Peach Purée (%)	Jujube Powder (%)	Sugar (%)	Stevia (%)	Water (%)	Citric Acid (%)
Control	63.00	-	15.99	-	21.00	0.01
SR5	58.00	5.00	15.99	-	21.00	0.01
SR10	53.00	10.00	15.99	-	21.00	0.01
SR15	48.00	15.00	15.99	-	21.00	0.01
SZJ	63.00	15.99	-	-	21.00	0.01
SSR	68.50	-	-	8.49	23.00	0.01

**Table 2:** TSS (%), ash (%), moisture (%) content, pH, titratable acidity (TTA) (% citric acid), and initial temperature (°C) of studied sorbet matrices.

Sorbet Variations	TSS (%)	Ash (%)	Moisture (%)	TTA (%)	pH	*t*, °C
Control	26.53 ± 0.25 ^bc^	5.21 ± 3.05 ^a^	69.27 ± 1.77 ^c^	0.21 ± 0.01 ^a^	3.37	−4.5
SR5	29.00 ± 0.72 ^b^	3.77 ± 0.18 ^ab^	64.11 ± 3.45 ^d^	0.19 ± 0.01 ^a^	3.62	−4.7
SR10	31.93 ± 0.25 ^a^	2.22 ± 1.28 ^ab^	64.92 ± 1.74 ^cd^	0.19 ± 0.02 ^a^	3.60	−5.7
SR15	33.03 ± 0.98 ^a^	0.36 ± 0.20 ^b^	63.77 ± 0.34 ^d^	0.18 ± 0.01 ^a^	3.64	−6.4
SZJ	24.84 ± 1.70 ^c^	1.09 ± 0.47 ^b^	74.27 ± 0.32 ^b^	0.27 ± 0.11 ^a^	3.88	−4.2
SSR	17.50 ± 1.01 ^d^	0.51 ± 0.38 ^b^	80.21 ± 0.56 ^a^	0.18 ± 0.02 ^a^	3.58	−3.2

Different letters in the same column indicate statistically significant differences (*p* < 0.05), according to ANOVA (one-way) and the Tukey test. SR5, SR10, SR15, SZJ, SSR—as presented in Table 1.

**Table 3:** Vitamin C (mg %) content of studied sorbet matrices.

Sorbet Variations	Vit C, mg%
control	0.04 ± 0.005 ^c^
SR5	0.08 ± 0.01 ^b^
SR10	0.10 ± 0.02 ^b^
SR15	0.15 ± 0.021 ^a^
SZJ	0.14 ± 0.01 ^a^
SSR	0.17 ± 0.01 ^a^

Different letters in the same column indicate statistically significant differences (*p* < 0.05), according to ANOVA (one-way) and the Tukey test. SR5, SR10, SR15, SZJ, SSR—as presented in Table 1.

**Table 4:** Nutritional data of sorbet matrices.

Sorbet Variations, 100 g	Proteins, g	Carbohydrates, g	Sugars, g	Fiber, g	Fat, g	Energy, kcal
SR5	0.24	22.66	20.53	1.57	1.62	106.65
SR5	0.48	26.20	23.73	1.65	1.49	121.39
SR10	0.73	29.70	26.91	1.74	1.37	136.54
SR15	0.97	33.30	30.13	2.07	1.49	158.39
SZJ	1.08	19.70	15.92	2.24	1.62	101.85
SSR	1.48	8.98	4.98	1.69	2.32	68.60

**Table 5:** Overrun (%) studied sorbet matrices.

Sorbet Variations	Overrun (%)
Control	10.23 ± 0.06 ^c^
SR5	8.11 ± 0.08 ^d^
SR10	11.06 ± 0.03 ^b^
SR15	11.02 ± 0.04 ^b^
SZJ	8.11 ± 0.08 ^d^
SSR	12.32 ± 0.05 ^a^

Different letters in the same column indicate statistically significant differences (*p* < 0.05), according to ANOVA (one-way) and the Tukey test. SR5, SR10, SR15, SZJ, SSR—as presented in Table 1.

**Table 6:** CIELAB color spectra of sorbet matrices.

Pudding Formulations	L	a	b	c	h
control	55.73 ± 3.74 ^a^	15.04 ± 1.40 ^a^	29.56 ± 1.55 ^a^	33.17 ± 1.94 ^a^	63.07 ± 1.34 ^a^
SR5	41.65 ± 4.25 ^b^	13.41 ± 0.46 ^a^	26.97 ± 3.01 ^a^	30.13 ± 2.83 ^a^	63.40 ± 2.15 ^a^
SR10	57.91 ± 4.27 ^a^	15.09 ± 1.63 ^a^	32.16 ± 6.66 ^a^	35.55 ± 6.68 ^a^	54.45 ± 2.78 ^a^
SR15	46.98 ± 6.63 ^ab^	13.38 ± 1.28 ^a^	27.08 ± 2.91 ^a^	30.22 ± 3.03 ^a^	63.65 ± 1.85 ^a^
SZJ	42.13 ± 3.18 ^b^	14.72 ± 0.75 ^a^	29.95 ± 2.26 ^a^	33.37 ± 2.35 ^a^	63.79 ± 0.58 ^a^
SSR	42.13 ± 3.81 ^b^	12.28 ± 1.02 ^a^	28.78 ± 3.35 ^a^	31.10 ± 3.18 ^a^	66.82 ± 1.03 ^a^

Different letters in the same column indicate statistically significant differences (*p* < 0.05), according to ANOVA (one-way) and the Tukey test. SR5, SR10, SR15, SZJ, SSR—as presented in Table 1.

**Table 7:** Microbial count of sorbet matrices.

Sorbet Variations	YM, CFU/mL	AMM, CFU/mL
Control sample	2100	1350
SR5	700	850
SR10	1100	1100
SR15	800	650
SZJ	5400	5850
SSR	3800	3050

**Table 8:** Water-holding capacity (WHC) (%) of studied sorbet matrices.

Scheme Variations	WHC (%)
Control	19.42 ± 0.52 ^a^
SR5	8.45 ± 1.25 ^b^
SR10	4.15 ± 0.03 ^c^
SR15	0.67 ± 0.06 ^d^
SZJ	0.47 ± 0.04 ^d^
SSR	17.46 ± 1.15 ^a^

Different letters in the same column indicate statistically significant differences (*p* < 0.05), according to ANOVA (one-way) and the Tukey test. SR5, SR10, SR15, SZJ, SSR—as presented in Table 1.

**Table 9:** Sensory evaluation of sorbet variations.

Attribute/Sample	Control	SR5	SR10	SR15	SZJ	SSR
**Appearance**						
Pink color	6.5 ± 1.08 ^a^	1.1 ± 0.73 ^d^	2.1 ± 0.57 ^bc^	2.4 ± 0.69 ^b^	1.2 ± 0.42 ^cd^	2.3 ± 0.67 ^b^
Orange color	2.8 ± 1.03 ^d^	9.9 ± 0.74 ^c^	10.3 ± 0.67 ^c^	10.7 ± 0.48 ^c^	12.0 ± 0.47 ^b^	13.3 ± 0.48 ^a^
Icy surface	5.5 ± 1.08 ^a^	3.6 ± 0.69 ^b^	2.3 ± 0.48 ^c^	2.3 ± 0.48 ^c^	2.6 ± 0.51 ^c^	5.3 ± 0.49 ^a^
Uniformity of color	12.0 ± 0.74 ^bc^	13.3 ± 0.67 ^a^	11.8 ± 0.63 ^bc^	12.2 ± 0.42 ^b^	11.3 ± 0.48 ^c^	13.1 ± 0.57 ^a^
Hard to spoon	8.7 ± 0.82 ^a^	4.8 ± 1.3 ^b^	4.4 ± 0.52 ^b^	2.2 ± 0.63 ^c^	1.2 ± 0.42 ^c^	2.1 ± 0.73 ^c^
Slimy to touch	7.0 ± 1.50 ^a^	7.0 ± 1.05 ^a^	4.8 ± 0.63 ^b^	3.3 ± 0.48 ^c^	2.3 ± 0.67 ^cd^	2.0 ± 0.81 ^d^
**Aroma**						
Fruity	7.1 ± 1.90 ^a^	7.1 ± 1.19 ^a^	3.7 ± 0.48 ^b^	4.7 ± 0.48 ^b^	0.6 ± 0.51 ^c^	8.4 ± 0.51 ^a^
Rancid	0.0 ± 0.0 ^a^	0.0 ± 0.0 ^a^	0.0 ± 0.0 ^a^	0.0 ± 0.0 ^a^	0.0 ± 0.0 ^a^	0.0 ± 0.0 ^a^
Earthy	0.0 ± 0.0 ^b^	0.0 ± 0.0 ^b^	0.0 ± 0.0 ^b^	0.0 ± 0.0 ^b^	0.0 ± 0.0 ^b^	2.4 ± 0.51 ^a^
Sweet	2.5 ± 0.85 ^a^	2.5 ± 0.85 ^a^	1.3 ± 0.48 ^b^	2.7 ± 0.48 ^a^	0.3 ± 0.48 ^c^	1.5 ± 0.53 ^b^
Fermented	1.0 ± 0.66 ^b^	1.0 ± 0.67 ^b^	2.4 ± 0.52 ^a^	0.8 ± 0.42 ^b^	2.5 ± 0.53 ^a^	1.0 ± 0.0 ^b^
**Flavor**						
Sweet	12.4 ± 0.84 ^b^	14.3 ± 0.84 ^a^	14.9 ± 0.31 ^a^	14.3 ± 0.48 ^a^	9.5 ± 0.53 ^c^	12.4 ± 0.51 ^b^
Sour	8.1 ± 0.99 ^a^	0.3 ± 0.48 ^e^	2.7 ± 0.67 ^d^	0.6 ± 0.51 ^e^	6.1 ± 0.74 ^b^	4.8 ± 0.63 ^c^
Fruity	10.2 ± 0.78 ^b^	11.4 ± 0.51 ^a^	4.3 ± 0.48 ^e^	5.4 ± 0.51 ^d^	7.2 ± 1.03 ^c^	6.5 ± 0.85 ^c^
Bitter	0.0 ± 0.0 ^e^	2.1 ± 0.56 ^d^	2.9 ± 0.57 ^c^	3.7 ± 0.48 ^b^	7.3 ± 0.67 ^a^	8.0 ± 0.67 ^a^
Tasteless	0.0 ± 0.0 ^a^	0.0 ± 0.0 ^a^	0.0 ± 0.0 ^a^	0.0 ± 0.0 ^a^	0.0 ± 0.0 ^a^	0.0 ± 0.0 ^a^
**Mouthfeel**						
Melting	13.3 ± 0.94 ^a^	13.3 ± 0.48 ^a^	12.0 ± 0.67 ^b^	11.8 ± 0.63 ^b^	12.3 ± 0.48 ^b^	13.2 ± 0.42 ^a^
Ice cube-like	0.6 ± 0.51 ^a^	0.6 ± 0.51 ^a^	0.7 ± 0.48 ^a^	0.7 ± 0.48 ^a^	0.5 ± 0.52 ^a^	0.0 ± 0.0 ^a^
Teeth numbing	2.4 ± 0.96 ^b^	1.3 ± 0.48 ^c^	1.0 ± 0.47 ^c^	0.7 ± 0.48 ^c^	0.6 ± 0.52 ^c^	7.6 ± 0.69 ^a^
Salivating	8.3 ± 0.94 ^a^	9.1 ± 0.56 ^a^	6.8 ± 0.42 ^b^	6.9 ± 0.57 ^b^	6.1 ± 0.57 ^b^	3.7 ± 0.48 ^c^
Soft	8.4 ± 0.96 ^c^	12.1 ± 0.56 ^b^	11.9 ± 0.57 ^b^	12.2 ± 0.63 ^b^	12.3 ± 0.48 ^ab^	13.1 ± 0.31 ^a^
Easy to dissolve	9.7 ± 1.15 ^c^	11.9 ± 0.57 ^b^	12.9 ± 0.73 ^a^	12.1 ± 0.56 ^ab^	12.3 ± 0.48 ^ab^	12.5 ± 0.53 ^ab^
Uneven consistency	0.8 ± 0.78 ^a^	0.4 ± 0.51 ^a^	0.5 ± 0.71 ^a^	0.4 ± 0.51 ^a^	0.3 ± 0.48 ^a^	0.0 ± 0.0 ^a^
**Aftertaste**						
Sweet	11.6 ± 0.84 ^b^	13.1 ± 0.57 ^a^	12.3 ± 0.48 ^ab^	12.8 ± 0.42 ^a^	10.2 ± 0.42 ^c^	5.3 ± 0.83 ^d^
Sour	1.3 ± 0.67 ^bc^	0.0 ± 0.0 ^e^	1.0 ± 0.0 ^cd^	0.3 ± 0.48 ^de^	4.4 ± 1.17 ^a^	1.9 ± 0.74 ^b^
Fruity	9.4 ± 0.69 ^a^	0.6 ± 0.51 ^c^	0.6 ± 0.51 ^c^	5.2 ± 0.42 ^b^	1.0 ± 0.0 ^c^	1.0 ± 0.0 ^c^
Bitter	0.0 ± 0.0 ^d^	0.0 ± 0.0 ^d^	0.7 ± 0.48 ^c^	0.7 ± 0.48 ^c^	6.3 ± 0.68 ^b^	8.5 ± 0.52 ^a^
Salivating	4.5 ± 0.70 ^c^	6.2 ± 0.63 ^a^	5.5 ± 0.52 ^ab^	5.4 ± 0.52 ^bd^	1.9 ± 0.31 ^b^	0.8 ± 0.42 ^e^

Different letters in the same row indicate statistically significant differences (*p* < 0.05), according to ANOVA (one-way) and the Tukey test. SR5, SR10, SR15, SZJ, SSR—as presented in Table 1.

## Data Availability

The data presented in this study are available on request from the corresponding author.
